# Melanoma: Stem cells, sun exposure and hallmarks for carcinogenesis, molecular concepts and future clinical implications

**DOI:** 10.4103/1477-3163.62141

**Published:** 2010-04-01

**Authors:** Athanassios Kyrgidis, Thrasivoulos-George Tzellos, Stefanos Triaridis

**Affiliations:** 1 Department of Otolaryngology, Head & Neck Surgery, School of Medicine, Aristotle University of Thessaloniki, Thessaloniki, Greece; 2 Department of Pharmacology, School of Medicine, Aristotle University of Thessaloniki, Thessaloniki, Greece

**Keywords:** Biomarkers, carcinogenesis, melanoma, melanomagenesis, stem cells, sunlight, ultra violet light exposure

## Abstract

**Background:**

The classification and prognostic assessment of melanoma is currently based on morphologic and histopathologic biomarkers. Availability of an increasing number of molecular biomarkers provides the potential for redefining diagnostic and prognostic categories and utilizing pharmacogenomics for the treatment of patients. The aim of the present review is to provide a basis that will allow the construction–or reconstruction–of future melanoma research.

**Methods:**

We critically review the common medical databases (PubMed, EMBASE, Scopus and Cochrane CENTRAL) for studies reporting on molecular biomarkers for melanoma. Results are discussed along the hallmarks proposed for malignant transformation by Hanahan and Weinberg. We further discuss the genetic basis of melanoma with regard to the possible stem cell origin of melanoma cells and the role of sunlight in melanoma carcinogenesis.

**Results:**

Melanocyte precursors undergo several genome changes –UV-induced or not– which could be either mutations or epigenetic. These changes provide stem cells with abilities to self-invoke growth signals, to suppress antigrowth signals, to avoid apoptosis, to replicate without limit, to invade, proliferate and sustain angiogenesis. Melanocyte stem cells are able to progressively collect these changes in their genome. These new potential functions, drive melanocyte precursors to the epidermis were they proliferate and might cause benign nevi. In the epidermis, they are still capable of acquiring new traits via changes to their genome. With time, such changes could add up to transform a melanocyte precursor to a malignant melanoma stem cell.

**Conclusions:**

Melanoma cannot be considered a “black box” for researchers anymore. Current trends in the diagnosis and prognosis of melanoma are to individualize treatment based on molecular biomarkers. Pharmacogenomics constitute a promising field with regard to melanoma patients' treatment. Finally, development of novel monoclonal antibodies is expected to complement melanoma patient care while a number of investigational vaccines could find their way into everyday oncology practice.

## BACKGROUND

Melanoma is the type of skin cancer with the highest death toll in the United States and Europe.[1] Even though melanoma was considered a rare cancer a century ago, the average lifetime risk for a person developing melanoma has now reached 1 in 50 in many Western populations.[2] Specifically, the lifetime risk for developing melanoma is 1/53 for men and women[3] in the United States, but has been reported to be as high as 1/25 for Australian males.[4] In the United States, the annual percent change for cutaneous melanoma in men and women was reported to be 2.4% for the period 1986-2006, thereby confirming melanoma as one of the malignancies that still exhibits significantly positive rates of increase;[3] 68,720 new cases of melanoma were expected to occur in the US while 8,650 patients are expected to have died from the disease in 2009.[5]

The promise of biomarkers of response for melanoma is enormous, but to date, this potential remains largely untapped.[6] Biomarkers are tumor- or host-related factors that correlate with tumor biological behavior and patient prognosis. Practically, a biomarker is any measurable diagnostic indicator that is used to assess the risk or presence of disease.[7]

Back in 2000, Hanahan and Weinberg reported the hallmarks of cancer.[8] In recent years, cancer research has been focusing on finding new biomarkers of potential prognostic value and on better understanding the pathophysiological processes of the disease, on improving current therapies or introducing novel treatment approaches.[9] Human cells, in order to transform to cancer cells, need to 1) provide growth signals—obtain growth self-sufficiency, 2) ignore growth-inhibitory signals, 3) avoid apoptosis, 4) replicate without limit, 5) sustain angiogenesis and 6) invade and proliferate.[9]

Apoptosis is the process of programmed cell death that occurs in multicellular organisms. Programmed cell death involves a series of biochemical events leading to a characteristic cell morphology and death. Apoptosis occurs via two interconnected pathways: the extrinsic (death receptor) and intrinsic (mitochondrial-dependent) pathways, both of which ultimately lead to the activation of effector-caspases (caspases-3, -6, -7), the final mediators of cell death,[10] PTEN, CDKN2A and APAF-1.[4]

Epigenetics refer to changes in phenotype (appearance) or gene expression caused by mechanisms other than changes in the underlying DNA sequence, hence the name epi-(Greek: over; above)-genetics. These changes may remain through cell divisions for the remainder of the cell's life and may also last for multiple generations. However, there is no change in the underlying DNA sequence of the organism; instead, non-genetic factors cause the organism's genes to behave differently. Epigenetic alterations include both DNA modification (methylation of CpG islands) and alteration in DNA packaging (histones). Over the past five years, more than 50 genes in melanoma have been shown to be dysregulated through epigenetic changes.[11] Epigenetic changes and also mutations, could be augmented by ultraviolet radiation (UVR). Extensive epidemiological evidence implicates intermittent intense exposure to UVR, especially during childhood, as a major risk factor in the etiology of melanoma.[12] The fact that the major risk factor for melanoma has long been appreciated and the range from benign nevus to advanced melanoma are readily visible and excisable, makes melanoma highly amenable to molecular analysis.[13] However, despite these advantages, the mechanisms by which sunlight induces melanomagenesis and melanoma cells acquire the capacity to metastasize are not adequately understood. This review aims to summarize what is currently known about the hal lmarks of melanoma but also to provide a basis that will allow the construction –or reconstruction – of future melanoma research.

## MATERIALS AND METHODS

We systematically reviewed the common medical databases (PubMed, EMBASE, Scopus and Cochrane CENTRAL) for studies reporting on molecular biomarkers for melanoma. Articles were not reviewed on the grounds of outcome or methodology. Rather, the systematic review process aimed to identify eligible articles to support the aim of this study. Results were critically appraised and classified according to the hallmarks for cell malignant transformation proposed by Hanahan and Weinberg, the genetic basis of melanoma, the stem cell cancer theory and the role of sunlight in carcinogenesis.

### Stem cells

While extensive progress has been made on the molecular mechanics of cancer, the exact population of cancer cells where these changes will occur has not been defined. Along with the progress of stem-cell biology, the possible existence of “cancer stem-cells”, that is rare cancer cells with indefinite potential for self-renewal that drive carcinogenesis has been proposed.[14] The recognition of a subpopulation of tumor stem cells (TSCs) in solid cancers has reinvigorated the field. These cells have the capacity to self-renew and give rise to more differentiated cell forms.[15]

**Figure 1:**
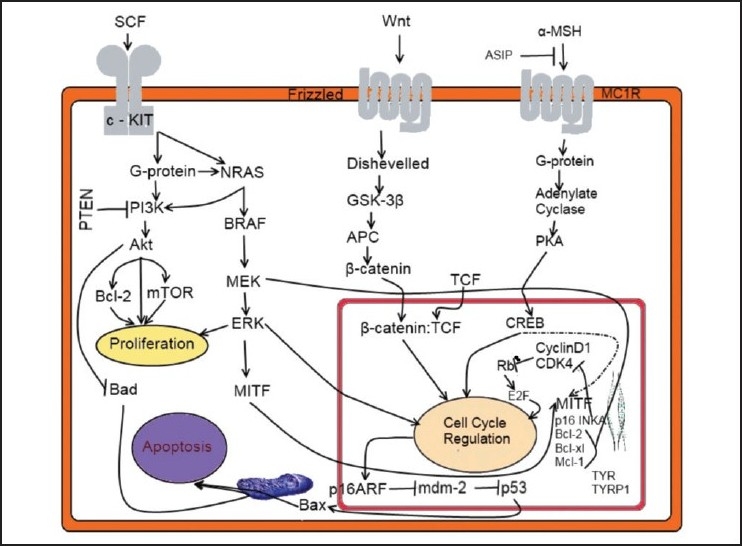
Melanoma intracellular signaling pathways: A simplified diagram of three of the major genetic networks involved in melanoma carcinogenesis. The SCF/c-KIT signaling network (NRAS, MAPK and PI3 Kinase/AKT pathways), the Wnt/Frizzled signaling network (APC, β-catenin), and the α-MSH/MC1R signaling network (PKA, CREB, MITF) which have been implicated in melanoma proliferation, apoptosis and cell cycle regulation. p16INKA and p14ARF are two separate tumor suppressors both of which are thought to contribute to senescence and tumor growth restriction. The p53 proapoptotic signaling network is also a major contributor to melanoma apoptosis and chemosensitivity and is regulated by many of the oncogenic melanoma pathways. →: designates a positive feedback ⊣: designates a negative feedback

TSCs have access to embryologic developmental programs, including the capacity to differentiate along multiple cell lineages.[15] To sustain and repair tissue, a considerable number of daughter cells, TSCs must maintain significant plasticity into adulthood.[16] Carcinogenesis is currently considered to be an aberrant developmental process.[4] Melanocytic cells appear to be primarily derived from the developing neural crest.[17] The melanocytic developmental process is very dependent on stem cell factor (SCF) and its receptor, c-KIT.[18] A major reservoir for melanocytic precursors is the hair follicle. With age and graying of the hair, residual melanocytic stem cell populations (CD133+) have been reported to decrease.[19] CD133+ cells constitute about 6% fetal skin cells but are only about 1% of adult cells.[20] It has been proposed that both melanocytes and mast cells in the skin are replenished from a common circulating CD133+ precursor.[15] Melanoma progression and metastasis is traditionally modeled as a stepwise process with the initial mutagenic event occurring in a melanocyte in the epidermis, with further mutation resulting in the proliferation passing through nevus and dysplastic nevus phases.[15] The cancer stem cell theory couples the idea that stem cells are responsible for cancer with the hypothesis that distinct mutations in signaling pathways are involved in carcinogenesis. Only long-time residents of the dermis/epidermis, most likely stem cells, have the ability to accumulate the number of necessary genetic hits that will result in melanoma development. According to the stem cell model, the initial mutations accumulate in a quiescent stem cell. When eventually, environmental signaling would activate the stem cell, due to these mutations, proliferation would not be appropriately controlled.[21] The neoplastic cells produced would attempt to follow normal melanocytic differentiation pathways including migration into the epidermis. In the epidermis, local growth factors would likely induce further proliferation. The stem cell component in the dermis would continue to expand, and some TSCs would inappropriately express germ cell pathways not only allowing for increased self-renewal but also driving genomic instability.[15] Therefore the target cell for the hallmarks of melanoma would likely be the CD133+ melanocytic stem cell [Figure 1].

### Sun exposure

UVR present in sunlight is an environmental human carcinogen. The toxic effects of UVR derived from natural sunlight or therapeutic artificial lamps are major concerns for human health. The main acute effects of UVR on normal human skin comprise erythema, tanning and local or systemic immunosuppression.[22]

At the molecular level, UVR exhibits deleterious effects on nucleic acids and proteins.[23] UVA mutates DNA indirectly via absorption by non-DNA endogenous sensitizers, which generate Reactive Oxygen Species(ROS) and lead to DNA damage.[24] UVB is considered to be more carcinogenic than UVA, as it directly causes two types of DNA lesions: cyclobutane pyrimidine dimers(CPDs), formed between adjacent thymine(T) or cytosine(C) residues, and 6-pyrimidine 4-pyrimidone photoproducts(6-4PP).[22,25] These UVB-induced lesions give rise to DNA mutations hallmarked by CT→T and CC→TT transitions, the so-called “UVB signature mutations”.[13] Definitive evidence for UVB signature mutations in melanoma genes has been surprisingly lacking in the literature.[13] Experimental studies in mice which developed malignant melanoma, demonstrated a role of UVB in inducing melanomagenesis.[26]

DNA damage is usually repaired by nucleotide excision repair (NER). NER is instrumental in removing DNA lesions caused by UVR.[23] Studies have examined polymorphisms in genes associated with DNA repair and identified significant association with xeroderma pigmentosum complementating group D(XPD, locus 19q13.2-3) gene and suggested that XPD gene polymorphisms might predispose to melanoma in the general population.[27]

Recent studies have also provided evidence for the presence of several genes whose expression could be altered in melanoma by epigenetic modulations.[13] It is not currently known if UVR can initiate melanomagenesis through an epigenetic mechanism.[13] In lung cancer, silencing of genes by hypermethylation has been thoroughly documented.[28–29] Rochette *et al.* recently reported that methylated cytosines are significantly more susceptible to CPD formation compared with unmethylated cytosines following UVB exposure.[30] Thus a possible linkage between epigenetic silencing or gene amplification and UVB exposure already exists for melanoma.

Aberrant proliferation of normal melanocytes, presumably in response to UVR, could result in the formation of benign or dysplastic nevi. In its radial growth phase melanoma exhibits the ability to grow intra-epidermally, followed by the ability to invade into the dermis in the vertical growth phase and culminating with metastasis. Only about half of the melanomas are known to arise from nevi, and progression can occur without going through these phases.[13] However, spontaneous DNA mutations have also been observed in several genes and are postulated to be involved at different phases of melanoma progression [Figure 1].

### Hallmarks for carcinogenesis

1) Provide growth signals – obtain growth self-sufficiency

#### NRAS

Marshall *et al*.[31] identified a gene with transforming activity in two different human sarcoma cell lines and identified this gene as a member of the RAS gene family and designated it NRAS.[32] Neuroblastoma RAS Viral Oncogene Homolog (NRAS; gene map locus 1p13.2) gene has been reported to be mutated in melanoma cell lines.[33] Subsequent studies validated the role of RAS in melanoma biology because activating NRAS mutations are relatively common(26%) in sporadic melanoma. NRAS is capable of inducing melanoma in CDKN2A-deficient mice and has been shown to play a key role in melanoma maintenance in murine models.[4] Although NRAS is now accepted to be a key oncogene in human melanoma, other members of the RAS family of proteins have a limited role in humans, regarding melanoma development. HRAS alterations have been reported to be present in Spitz nevi.[34] NRAS mutations have been reported to be more frequent on chronically sun-exposed sites.[4] However, NRAS mutations appear to be at least as common in congenital nevi as in melanomas[4,33,35–36] signifying that sun exposure is not necessary to induce the commonest NRAS mutations.

Given the sheer prevalence of RAS mutations in human cancer, direct and efficacious inhibition of RAS would be an ideal therapeutic goal. Farnesyltransferase inhibitors, such as tipifarnib and lonafarnib, block RAS activation by inhibiting post-translational farnesylation thereby preventing translocation of RAS to the plasma membrane and subsequent RAS signaling. Inhibition of RAS activity could also be leveraged to reduce chemoresistance. Preliminary data suggest that lonafarnib, together with cisplatin, may enhance melanoma apoptosis *in vitro* [Figure 1].[37]

#### BRAF

Davies *et al*.[38] identified V-Raf Murine Sarcoma Viral Oncogene Homolog B1(BRAF; gene map locus 7q34) somatic missense mutations in 66% of malignant melanomas and at lower frequency in a wide range of human cancers. All mutations were within the kinase domain, with a single substitution, V600E accounting for 80%.[38–40] RAS function is not required for the growth of cancer cell lines with the V600E mutation.[38,40] The authors suggested that since BRAF is a serine/threonine kinase that is commonly activated by somatic point mutation in human cancer, it may provide new therapeutic opportunities in malignant melanoma.[38] Oncogenic BRAF mutations lead to constitutive activation of the kinase activity of BRAF, which provides the cell with continuous growth signals in the absence of extracellular stimuli.[2] Mutagenic activation of the mitogen-activated protein kinase(MAPK) pathway is observed in up to 90% of melanomas and is most commonly achieved via mutations in NRAS or BRAF. Mutations in these two genes occur in a mutually exclusive manner.[4] A large series reported that in 57.5% of the tumors either BRAF or NRAS were mutated with a higher incidence in metastatic (66.3%) than in primary lesions (50.9%).[35] The lack of UVB signature mutations in both NRAS and BRAF suggest that the mitogen-activated protein kinase (MAPK) pathway components are not the primary targets of UVB.[13] As BRAF mutations occur at such a high rate in melanomas, targeted approaches against BRAF V600E represent an ideal approach. Sorafenib is a first-generation non-selective RAF inhibitor that has been shown to abrogate MAPK signaling biochemically and to harbor anti-melanoma effects *in vitro*.[41] Early clinical trials have failed to show significant efficacy with sorafenib as monotherapy in advanced melanoma.[4] While recent results with sorafenib in combination with carboplatin and paclitaxel have not been very promising,[42] in cases where pre-treatment pharmacogenomic screening for the expression of sorafenib targets was performed, specific benefits have been reported.[43] More specifically, high VEGF -R2 expression is associated with response to sorafenib, carboplatin, and paclitaxel combination treatment in melanoma, whereas high MEK expression is associated with resistance [Figure 1].[43]

#### MEK

MEK kinase (MITOGEN-ACTIVATED KINASE 1; MAP3K1; gene map locus 5q11.2), is a serine/threonine kinase that occupies a pivotal role in a network of phosphorylating enzymes integrating cellular responses to a number of mitogenic and metabolic stimuli, including insulin and many growth factors.[4,36]

A Phase I/II trial of PD0325901 (NCT00147550) enrolled 27 patients and demonstrated partial responses in 2/27 and stable disease in 5/27.[4] Unfortunately this trial was prematurely discontinued due to safety concerns, specifically ocular and neurological toxicity. MEK inhibition is a prime target for melanoma therapy given BRAF's downstream dependence on MEK for signal propagation [Figure 1]. Recently, melanoma cell lines harboring BRAF mutations were found to be markedly more sensitive to MEK inhibition than lines with NRAS mutations.[4,36] GSK1120212 is a potent and highly selective inhibitor of MEK activation and kinase activity.[44] The objective response rate, safety, and pharmacokinetics of GSK1120212 in BRAF mutation-positive melanoma subjects previously treated with a BRAF inhibitor is under investigation (NCT01037127).[44]

#### AKT

Phosphoinositide 3-kinases, or PI3Ks, generate specific inositol lipids implicated in the regulation of cell growth, proliferation, survival, differentiation, and cytoskeletal changes. One of the best characterized targets of PI3K lipid products is the protein kinase AKT(PKB; V-AKT MURINE THYMOMA VIRAL ONCOGENE HOMOLOG 1; PROTEIN KINASE B-ALPHA; gene map locus 14q32.3). Aziz *et al*.,[45] studied PI3K protein expression in 548 melanomas and 540 benign nevi and demonstrated that PI3K expression was higher in melanomas when compared to nevi, while it was significantly higher in those specimens from patients with metastases.[45] The PI3K/Akt3 signaling pathway is aberrantly activated in up to 70% of melanomas and has been implicated in tumor progression and chemoresistance.[4] Although direct targeting of Akt3 has the potential of being an effective therapeutic approach in melanoma, the ATP-binding regions are highly conserved between the three isoforms of Akt (Akt1, Akt2, Akt3) thus making it difficult to specifically inhibit Akt3 without also inhibiting the other isoforms and enduring the resulting toxicity.[4] Thus, one approach that has been employed is to target direct substrates of Akt3, such as mTOR(FKBP12-RAPAMYCIN COMPLEX-ASSOCIATED PROTEIN 1; FRAP1; gene map locus 1p36.2). In fact, of the drugs currently being tested in PI3K pathway inhibition, mTOR inhibitors, namely Temsirolimus and Everolimus are the most advanced.

Preliminary data from the only completed Phase II trial (NCT00022464) of 33 patients with metastatic melanoma, monotherapy with Tensirolimus showed one partial response lasting two months with a median time-to-disease progression of 10 weeks.[46] More promising results were seen in the interim analysis of a recent two-stage Phase II trial[47] using everolimus as monotherapy in patients with Stage IV melanoma. Of the 20 patients studied, 35% experienced stable disease at 16 weeks [Figure 1].[48]

#### KIT

The tyrosine kinase receptor c-KIT (KIT HARDYZUCKERMAN 4 FELINE SARCOMA VIRAL ONCOGENE HOMOLOG;STEM CELL FACTOR RECEPTOR;SCFR; gene map locus 4q12) and its ligand, KITLG (KIT LIGAND;STEM CELL GROWTH FACTOR;SCF; gene map locus 12q22), function in hematopoiesis, melanogenesis, and gametogenesis.[49] It has been proposed that c-KIT can be both an oncogene and therapeutic target in certain melanoma types (mucosal, acral, and chronically sun-exposed melanomas.), via multiple melanocytic signaling pathways.[4,50] Imatinib mesylate (Gleevec^®^) is a receptor tyrosine kinase inhibitor that inhibits BCR-ABL, as well as c-KIT,[51] and has been FDA-approved for treating chronic myelogenous leukemia and gastrointestinal stromal tumors. Imatinib monotherapy is administered in patients with mucosal, acral or lentiginous melanoma,[52] metastatic melanoma[53] and melanoma that cannot be removed by surgery[54] in ongoing clinical trial settings. Another ongoing trial, examines whether nilotinib (Tasigna^®^) is more efficacious than dacarbazine in the treatment of metastatic inoperable melanomas harboring c-KIT mutations[55] [Figure 1].

### Ignore growth inhibitory signals

#### RB

The retinoblastoma gene RB (RETINOBLASTOMA; gene map locus 13q14.1-q14.2) was the first tumor suppressor gene cloned, and is a negative regulator of the cell cycle through its ability to bind the transcription factor E2F and repress transcription of genes required for S phase.[8] No studies have systematically examined the status of RB1 in melanoma. It appears that mutations in CDKN2A, CDK4 and RB1 occur in a mutually exclusive manner.[2]

#### CDKN2A

The CDKN2A (CYCLIN-DEPENDENT KINASE INHIBITOR 2A;CDKN2A;CDKN2;gene map locus 9p21) gene encodes proteins that regulate two critical cell cycle regulatory pathways, the p53 pathway and the RB pathway. Through the use of shared coding regions and alternative reading frames, the CDKN2A gene produces two major proteins: p16INK4A, which is a cyclin dependent kinase inhibitor, and p14ARF, which binds the p53-stabilizing protein MDM2.[56] p16INK4A inhibits Cdk4/6-mediated phosphorylation of the retinoblastoma protein. In the hypophosphorylated state, RB binds and represses the E2F transcription factor and prevents G1→S transition. Alternatively, p14ARF directly prevents MDM2 from accelerating the degradation of p53 [Figure 1]. Loss of the CDKN2A locus negatively impacts on both the RB and p53 pathways. In addition to its effects on the p53 pathway, there is emerging evidence to suggest that p14ARF also possesses tumor-suppressive effects that are independent of p53. Ha *et al*., recently used a mouse model to demonstrate that p14ARF but not p53, suppressed early melanomagenesis and operated independent of p53 to induce melanocyte senescence.[57] This added benefit of p14ARF loss may help explain why CDKN2A, as opposed to p53, is preferentially abrogated in melanoma.[4] CDKN2A expression changes were identified in the vast majority of melanomas through mutation, deletion or promoter hypermethylation of CDKN2A gene.[2] CDKN2A harbors mutations in melanoma at dipyrimidine sites but many of these genetic alterations are not UVB signature mutations.[13] An ongoing trial is aimed to identify gene mutations in patients with melanoma and in families with a history of hereditary melanoma and monitors for CDKN2A mutations.[58] Restoring function of a lost or defective gene in cancer has generally proven to be more difficult than blocking a hyperfunctional gene; thus it is not surprising that most efforts in targeted melanoma therapy have focused on suppression of oncogene signaling rather than TSG restoration. Notably, hypermethylation of the CDKN2A promoter region has been shown to lead to loss of p16 expression in 19% of primary and 33% metastatic melanomas.[59] DNA methyltransferase(DNMT) inhibitors namely 5-azacytidine, 5-aza-20-deoxycytidine(decitabine), fazarabine, and dihydro-5-azacytidine have been extensively studied. These agents when phosphorylated are incorporated into DNA and become covalently linked to DNMT, thereby preventing methylation. DNMT inhibitors have shown some promise in melanoma.[4] Promoter methylation status of the DNA repair enzyme methylguanine-DNA methyltransferase (MGMT; gene map locus 10q26) is being recorded as a secondary outcome in Phase II clinical trials of temozolomide and pegylated interferon alpha-2b[60] and temozolomide and everolimus[61] in melanoma patients.

#### CDK4

Cyclin-dependent kinase-4(CDK4; gene map locus 12q14) is a protein-serine kinase involved in the cell cycle. Human cell division is regulated primarily at the G1→S or the G2→M boundaries within the cell cycle. The sequential activation of cyclin-dependent kinases and their subsequent phosphorylation of critical substrates promote orderly progression through the cell cycle. The complexes formed by CDK4 and the D-type cyclins are involved in the control of cell proliferation during the G1 phase. CDK4 is inhibited by p16INK4A. Given that p16INK4A needs to directly interact with the cyclin–CDK complex in order to inhibit its protein kinase activity, changes in CDK4 that render it resistant to p16INK4A mimic p16INK4A loss [Figure 1]. In fact, both somatic and germline mutations in CDK4 have been detected in melanoma cell lines.[4] P276-00 is a flavone that inhibits cyclin-dependent kinases, and has been recently proposed as a novel antineoplastic agent.[62] ENVER is an ongoing trial that evaluates the efficacy of P276-00 in subjects with advanced malignant melanoma positive for CCND1 expression.[63]

#### CCND1

CCND1 (CYCLIN D1;B-CELL CLL/LYMPHOMA 1;BCL1;B-CELL LEUKEMIA 1;gene map locus 11q13) is the regulatory subunit of a holoenzyme that phosphorylates and inactivates the RB protein and promotes progression through the G1→S phase of the cell cycle in a manner dependent on CDKs. In addition, CCND1 is involved in a number of cell-cycle CDK-dependent and independent functions. CCND1 associates with and regulates transcription factors, coactivators, and corepressors that govern histone acetylating and chromatin-remodeling-proteins. CCND1 also has roles in cellular growth, metabolism, and cellular differentiation. Amplification or overexpression of CCND1 plays pivotal roles in the development of several human cancers, including parathyroid adenoma, breast cancer, colon cancer, lymphoma, melanoma, and prostate cancer.[8] Oncogenic RAS has been shown to positively impact the cell cycle by upregulating CCND1 through MAPK signaling[64] thereby serving as a link between the MAPK and p16-cyclin D/CDK4-RB pathways [Figure 1]. CCND1 amplifications were noted to occur more frequently on melanomas that arose on skin with chronic sun-induced damage, a subtype that rarely possesses BRAF or NRAS mutations.[65] Further to ENVER,[63] CCND1 and MAPK expression constitute secondary outcomes for clinical trials of tanespimycin[66] and sorafenib[67] in melanoma patients.

#### PTEN

Early loss-of-heterozygosity studies suggested the presence of a chromosome 10q locus lost early in tumor progression.[68] PTEN (PHOSPHATASE AND TENSIN HOMOLOG; gene map locus 10q23.31) was one of the putative TSGs on Chromosome 10 and had been shown to be mutated in gliomas, breast, prostate, and kidney cancers and melanomas.[4] PTEN mutations have been identified at overall frequencies of > 10% in uncultured melanomas and 30–50% in cell lines. PTEN is a negative regulator of the PI3K signaling pathway[69] and inactivation of PTEN by deletion or mutation leads to constitutive activation of this pathway [Figure 1]. PTEN mutations are found only rarely in primary melanomas,[70] suggesting that activation of the PI3K pathway may be responsible for the late processes in melanoma development, mainly invasion and metastasis.

Wang *et al*.,[71] studied samples from 59 melanomas. PTEN mutations were found in 56% of the melanomas, and 91% of the melanomas with mutations had one to four UVB-signature mutations. The authors stated that these data provide direct molecular evidence of UV involvement in melanoma induction in humans.[71] PTEN has been identified as a likely target for epigenetic silencing, because its expression is lost or decreased in up to 50% of melanomas, even in the absence of demonstrable mutations.[72] DNMT inhibitors can be used to target this type of epigenetic silencing. An ongoing trial[73] which examines how well MEK inhibitor AZD6244 works in treating patients with Stage III or Stage IV melanoma that cannot be removed by surgery has PTEN status in its secondary outcomes.

#### β-Catenin

Beta-catenin (CTNNB; gene map locus 3p22-p21.3) is an adherens junction protein and a transcriptional coactivator. Adherens junctions mediate adhesion between cells, communicate signals that neighboring cells are present and anchor the actin cytoskeleton; thus they regulate normal cell growth and behavior. The canonical Wnt signaling pathway stabilizes β-catenin, with MITF as a critical downstream target [Figure 1]. Disruption of the canonical Wnt pathway abrogated growth of melanoma cells, and constitutive overexpression of MITF rescued the growth suppression.[74] Functional analyses demonstrated that β-catenin immortalized melanocytes by repressing p16INKA expression, thereby bypassing the need for CDKN2A deletion.[75]

#### Avoid apoptosis

Development as well as maintenance of many adult tissues is achieved by several dynamically regulated processes that include cell proliferation, differentiation, and programmed cell death. This alternative senescence barrier in melanocytes is likely to involve p53, which is strongly up-regulated in p16INK4A-deficient melanocytes that have reached senescence.[76] The frequency of p53 mutations in melanoma derived specimens is reported to be 5–25%[2] which is relatively low compared with many other cancers, and may be explained by the frequent loss of p14ARF in melanoma through CDKN2A mutations and deletions. p14ARF is a positive regulator of p53, and loss of p14ARF function may have the same effect as loss of p53.

#### APAF-1

APAF-1 (APOPTOTIC PROTEASE ACTIVATING FACTOR 1; gene map locus 12q23), plays an essential role in apoptosis. In the presence of cytochrome C and dATP, APAF1 assembles into an oligomeric apoptosome, which is responsible for activation of procaspase-9 and maintenance of the enzymatic activity of processed caspase-9.[77] Soengas *et al*.,[78] showed that metastatic melanomas often lose APAF1 expression. The loss of APAF1 expression is accompanied by allelic loss in metastatic melanomas, which could be recovered in melanoma cell lines by treatment with the DNMT-inhibitor decitabine. APAF1-negative melanomas were documented to be consistently chemoresistant and unable to execute the classic apoptotic program in response to p53-activation. Restoring physiologic levels of APAF1 through gene transfer or decitabine treatment resulted in a 20-fold increase in APAF-1 expression, markedly enhanced chemosensitivity and rescued the apoptotic defects associated with APAF1 loss. The authors[78] concluded that APAF1 is inactivated in metastatic melanomas, leading to defects in the execution of apoptotic cell death. Using the DNMT inhibitor, they were able to restore APAF-1 levels, apoptosome function and chemosensitivity. Thus APAF-1 expression reversal via decitabine administration serves as proof-of-principle for DNMT inhibitors.[4] Decitabine is tested in melanoma either as combined treatment or as monotherapy as well as in metastatic melanoma as combined treatment.[79]

#### Bcl-2 gene family

The Bcl-2 network is thought to be one of the most crucial regulators of melanoma cell apoptosis.[80] At the core of the Bcl-2 network lives a family of anti-and proapoptotic proteins that function to regulate and execute the core mitochondrial pathway of apoptosis.[81] Proper regulation requires that a delicate balance is maintained between proapoptotic members of the Bcl-2 family (Bax, BAK, BAD, BID, Bim, NOXA, PUMA) and the antiapoptotic members (Bcl-2, Bcl-xL, Mcl-1, BCL-w, and A1).[4,81] Many of the signaling pathways responsible for tumor growth and progression have also been shown to possess profound downstream effects on the regulation of the Bcl-2 apoptotic network.

### Antiapoptotic Bcl-2 members

#### Bcl-2

Both effector and repressor genes exist within each mammalian cell death pathway. Bcl-2 (B-CELL CLL/LYMPHOMA 2; ONCOGENE B-CELL LEUKEMIA 2; gene map locus 18q21.3) is one such mammalian gene that has been identified; it functions as a repressor of programmed cell death. Bcl-2 which is the founding member of the Bcl-2 family of proteins, was first identified at the chromosomal breakpoint of t (14;18) in follicular B-cell lymphoma and found to block cell death following a variety of toxic insults.[82–84] High levels of Bcl-2 expression have been found in melanoma and melanocytes, providing a likely explanation for the resistance of melanocytes and melanoma to apoptosis induced by both physiological and therapeutic stimuli.[85]

Experimental data have shown that antisense suppression of Bcl-2 led to decreased melanoma cell survival and increased sensitivity to chemotherapy. Oblimersen, an 18-base antisense oligonucleotide that mediates cleavage of Bcl-2 mRNA, when supplemented to dacarbazine significantly improved multiple clinical outcomes in patients with advanced melanoma and increased overall survival in patients with normal baseline serum LDH.[86] In a meta-analysis, progression-free survival, response and durable response rates showed a highly significant difference favoring dacarbazine oblimersen while a nearly significant trend was noted in the overall survival. All efficacy parameters significantly favored dacarbazine-oblimersen in patients with normal baseline LDH.[87] Today, oblimersen (Genasense®) is studied in patients with advanced melanoma as combined treatment or monotherapy.[88]

#### Bcl-x

Other antiapoptotic Bcl-2 members might serve as a substitute for Bcl-2 once it is lost. Boise *et al*.,[89] isolated a Bcl-2-related gene, which they designated Bcl-X(Bcl-2-RELATED GENE;BCLX;BCLXL;BCLXS), and showed that it can function as a Bcl-2-independent regulator of programmed cell death (apoptosis). Alternative splicing resulted in two distinct Bcl-X mRNAs. The protein product of the larger mRNA (Bcl-xL) was similar in size and predicted structure to Bcl-2. When transfected into an IL3-dependent cell line, it inhibited cell death upon growth factor withdrawal at least as satisfactorily as Bcl-2.[89] Unexpectedly, the smaller mRNA species (Bcl-xS) was found to encode a protein that inhibited the ability of Bcl-2 to enhance the survival of growth factor-deprived cells. *In vivo*, Bcl-xS mRNA is expressed at high levels in cells that undergo a high rate of turnover, such as developing lymphocytes. In contrast, Bcl-xL is found in tissues containing long-lived post-mitotic cells, such as adult brain. These data suggested that Bcl-x plays an important role in both positive and negative regulation of programmed cell death.[89] Tumor cells are able to switch expression from Bcl-2 to Bcl-xL,[90] and in many cases Bcl-2 and Bcl-xL are expressed in a reciprocal fashion.[91] A bispecific Bcl-2/Bcl-xL antisense oligonucleotide 4625 has been manufactured which was shown to induce apoptosis in up to two-thirds of melanoma cells.[92]

#### Mcl-1

Mcl-1(MYELOID CELL LEUKEMIA 1; gene map locus 1q21) is a potent multidomain antiapoptotic protein of the Bcl-2-family that heterodimerizes with other Bcl-2-family members to protect against apoptotic cell death [Figure 1].[93] Mcl-1 is highly expressed in primary as well as advanced melanoma.[94] Multiple studies have now demonstrated that resistance to a variety of traditional and targeted chemotherapeutic agents is largely mediated by Mcl-1 overexpression.[95] Mcl-1 possesses qualities that distinguish it from other antiapoptotic Bcl-2 members. Whereas most antiapoptotic Bcl-2 members show little selectivity in their inhibition of proapoptotic members BAK and Bax, Mcl-1 has been shown to have a uniquely high specificity for suppressing apoptosis induced by BAK but not Bax.[96] Although Mcl-1 inhibitors may generally increase tumor-cell sensitivity to a variety of traditional chemotherapeutic agents, their unique relationship with the proteasome inhibitor class of chemotherapeutic agents may prove especially beneficial.[4,97] Like BAK, NOXA possesses a BH3 domain that has a high specificity for binding with Mcl-1. When activated by various stimuli, NOXA binds to Mcl-1, thereby freeing BAK from the BAK-Mcl-1 complex and promoting apoptosis.[97] Since bortezomib (Velcade®) simultaneously induces the proapoptotic Bcl-2 member, NOXA, and antiapoptotic Mcl-1, Mcl-1 inhibition is an attractive means for increasing the efficacy of bortezomib. Bortezomib led to increased MYC protein function and NOXA expression was only increased when c-Myc was bound to its recognition sites in the NOXA promoter.[95] Transduction of c-Myc into normal melanocytes, which are resistant to bortezomib, resulted in NOXA expression and newfound chemosensitivity to bortezomib. Similarly, transduction of ectopic c-Myc into melanoma cells led to increased response to lower doses of bortezomib and enhanced the extent of cell death by twofold.[4]

### Proapoptotic Bcl-2 members in melanoma

#### BAK

The functional analysis of BAK (Bcl-2 ANTAGONIST KILLER 1; gene map locus 6p21.3-p21.2), which promotes cell death and counteracts the protection from apoptosis provided by Bcl-2 was described in 1995.[98–99] Chittenden *et al*.,[99] found that enforced expression of BAK induced rapid and extensive apoptosis of serum-deprived fibroblasts. This suggested that BAK may be directly involved in activating the cell death machinery. Kiefer *et al*.,[98] pointed out that, like Bax, the BAK gene product primarily enhances apoptotic cell death following an appropriate stimulus. Unlike Bax, however, BAK can inhibit cell death in an Epstein-Barr virus transformed cell line.

#### Bax

The proapoptotic Bax (Bcl-2-ASSOCIATED X PROTEIN; gene map locus 19q13.3-q13.4) protein, induces cell death by acting on mitochondria [Figure 1]. Oltvai *et al*.,[100] showed that Bcl-2 associates *in vivo* with Bax. Bax shows extensive amino acid homology with Bcl-2 and forms homodimers and heterodimers with Bcl-2 *in vivo*. When Bax predominates, programmed cell death is accelerated, and the death repressor activity of Bcl-2 is countered. The ratio of Bcl-2 to Bax has been suggested[100] to determine survival or death following an apoptotic stimulus.

#### MITF

MITF (MICROPHTHALMIA-ASSOCIATED TRANSCRIPTION FACTOR; gene map locus 3p14.1-p12.3) is a basic helix-loop-helix leucine-zipper protein that plays a role in the development of various cell types, including neural crest-derived melanocytes and optic cup-derived retinal pigment epithelial cells.[101] MITF has multiple downstream effects, which together define numerous aspects of normal pigment cell physiology. MITF directly promotes melanocyte and possibly melanoma cell survival by inducing the expression of the protooncogene, Bcl-2, as one of its transcriptional targets.[4] MITF is also a downstream target of β-catenin, a critical regulator of melanoma cell growth [Figure 1]. [102] Given that MITF plays a central role in melanocyte and tumor survival, further delineation of the genetic network that regulates and is regulated by MITF, is warranted.

### Replicate without limit

Human telomeres consist of many kilobases of the “TTAGGG” sequence together with various associated proteins. Small amounts of these terminal sequences are lost from the tips of the chromosomes in each S phase because of incomplete DNA replication, but *de novo* addition of TTAGGG repeats by the enzyme telomerase compensates for this loss. Many human cells progressively lose terminal sequence with cell division, a loss that correlates with the apparent absence of telomerase in these cells.[103]

#### TERT

Human telomerase (TERT; TELOMERASE REVERSE TRANSCRIPTASE; gene map locus 5p15.33) was recognized and reported to catalyze the addition of a 6-nucleotide repeating pattern “TTAGGG” to oligonucleotide primers containing human or telomeric repeat sequences.[104] It has been reported that TERT expression had increased from control skin to peritumoral skin, and then to benign melanocytic lesions and finally to melanoma, suggesting tumor progression. TERT showed higher values in the presence of some important histopathologic parameters related to poor prognosis in cutaneous melanoma such as ulceration, vascular invasion, satellites, high rates of mitosis, and in thicker tumors.[105]

#### MYC

The MYC (MYC AVIAN MYELOCYTOMATOSIS VIRAL ONCOGENE HOMOLOG; gene map locus 8q24.12-q24.13) proto-oncogene encodes a ubiquitous transcription factor involved in the control of cell proliferation and differentiation. Deregulated expression of MYC caused by gene amplification, retroviral insertion or chromosomal translocation is associated with carcinogenesis. Understanding of the function of MYC and its role in carcinogenesis was aided by the demonstration by Wu *et al*., that MYC has a direct role in induction of the activity of telomerase.[106]

### Sustain angiogenesis

#### PTN

PTN (PLEIOTROPHIN;HEPARIN-BINDING NEURITE GROWTH-PROMOTING FACTOR 1;NEGF1; gene map locus 7q33) is a secreted polypeptide growth factor that is expressed in a variety of established tumor cell lines and primary human tumor specimen and is a mitogen for fibroblasts, epithelial cells derived from bovine lens or human adrenal carcinoma and endothelial cells. PTN can induce the release of active proteolytic enzymes from endothelial cells, can induce tube formation of endothelial cells *in vitro*, and thus, serve as a tumor angiogenesis factor.[107] PTN has been studied for melanoma angiogenesis and metastasis.[107] Wu *et al*., reported that within primary melanomas, PTN immunoreactivity was associated with metastasis and decreased melanoma-related survival. Univariate analysis of PTN immunoreactivity predicted an increased risk for metastasis (relative risk 9.1).[108]

### Invade and proliferate

#### NEDD9

NEDD9 (NEURAL PRECURSOR CELL EXPRESSED, DEVELOPMENTALLY DOWNREGULATED 9; HUMAN ENHANCER OF FILAMENTATION 1; HEF1; gene map locus 6p25-p24) enhanced invasion *in vitro* and metastasis *in vivo* of both normal and malignant melanocytes, functionally interacted with focal adhesion kinase and modulated focal contact formation, and exhibited frequent robust overexpression in human metastatic melanoma when compared to primary melanoma. Comparative oncogenomics has enabled the identification and facilitated the validation of a highly relevant cancer gene governing metastatic potential in human melanoma.[109]

#### SLUG

SLUG (SNAIL, DROSOPHILA, HOMOLOG OF, 2; SNAI2; gene map locus 8q11) belongs to the Snail-family of zinc-finger transcription factors that share an evolutionarily conserved role in mesoderm formation in invertebrates and vertebrates. SLUG triggers epithelial-mesenchymal transitions and plays an important role in developmental processes.[110] Slug has been shown to be a requisite for the metastasis of the transformed melanoma cells.[111]

#### KISS1

KISS1 (KISS1 METASTASIS SUPPRESSOR; METASTIN; KISSPEPTIN; gene map locus 1q32) is a human metastasis suppressor gene that suppresses metastases of melanomas and breast carcinomas without affecting tumorigenicity. *In vitro* studies report that KISS1 expression may suppress the metastatic potential of malignant melanoma cells.[111–113] Loss of Sp1-coactivator protein DRIP130, which is encoded by human chromosome 6q16.3-q23, results in reduced KISS1 promoter activation in highly malignant melanoma cells. DRIP130 is a key regulator in KISS1 transactivation in normal tissue, and the loss of DRIP130 expression, as a result of the gross loss of human chromosome 6q16.3-q23, provokes increased tumor metastasis.[114]

#### E-cadherin

ECAD (CADHERIN 1; E-CADHERIN; gene map locus 16q22.1) is a specific calcium ion-dependent cell adhesion molecule. In mice it expresses its adhesive function during the preimplantation stage of development in epithelial cells, where it is concentrated in the intermediate junctions. Activation of SLUG expression plays an important role in down-regulation of E-cadherin and tumorigenesis of malignant melanomas.[115] Melanoma cells at initial stages of the disease show reduction or loss of E-cadherin expression, but recovery of its expression is frequently found at advanced phases.[116] Overexpression of E-cadherin leads to defective invasion of melanoma cells.[116]

### Melanin synthesis biomarkers

All biomarkers reported to this point have a ubiquitous role in human cells. Here we will discuss some biomarkers which are specific for melanocyte development and proliferation.

#### MC1R

Melanocyte stimulating hormone (MSH; melanotropin) and adrenocorticotropic hormone (ACTH) regulate pigmentation and adrenocortical function, respectively. They are products of the same gene, the proopiomelanocortin gene. MSH and ACTH bind to receptors that couple to heterotrimeric guanine nucleotide-binding proteins(G proteins) which activate adenylyl cyclise [Figure 1].[117] Using PCR with primers based on conserved areas of other members of 7-transmembrane G protein-linked receptors, Gantz *et al.,*[118] isolated several genes encoding an ‘orphan’ subfamily of receptors specific for melanocortins. One was identified as an alpha-MSH seven-pass transmembrane G-protein-coupled receptor, otherwise known as the melanocortin-1 receptor(MC1R;MELANOCYTE-STIMULATING HORMONE RECEPTOR; gene map locus 16q24.3).[117–118] Apart from its role as a key regulator of melanin synthesis the MC1R gene had been reported to exhibit potential immunogenicity.[119] The same authors[119] evaluated its expression in uveal melanoma. Their results demonstrated that MC1R was expressed by uveal melanoma cells to a significantly greater extent than other melanoma markers. MC1R was found in 95% of melanoma tissues tested, including one liver metastasis. Even though MC1R was mainly located intracellularly, its cell surface expression could be promoted by cytokines, such as interferon-gamma and tumor necrosis factor-alpha. These data supported MC1R as a new marker for the diagnosis of uveal melanoma and as a putative therapeutic target.

The unambiguous importance of the MC1R variants towards the population burden of melanoma which have been reported to differ among the investigated populations[120] have motivated an ongoing clinical trial.[121] Based on the finding that genetic variants of the melanocortin-1 receptor(MC1R) gene, associated with red hair and fair skin, have been shown to be associated with increased risk for melanoma, particularly those harboring BRAF mutations,[122] the investigators intend to focus on the study of recently discovered genetic variants associated with pigmentation. Furthermore, the investigators will study the relation of these variants with oncogenic mutations of melanoma in BRAF, RAS and KIT.[2]

#### ASIP

The agouti gene on mouse Chromosome 2 encodes a paracrine signaling molecule that causes hair follicle melanocytes to synthesize pheomelanin, a yellow pigment, instead of the black or brown pigment eumelanin. Sequence analysis demonstrated that the coding region of the human ASIP (AGOUTI SIGNALING PROTEIN; gene map locus 20q11.2) gene is 85% identical to that of the mouse gene and has the potential to encode a protein of 132 amino acids with a consensus signal peptide.[123] In the latter study, the authors[123] genotyped 746 participants. Carriage of the G allele was significantly associated with dark hair (odds ratio 1.8) and brown eyes (odds ratio 1.9) after adjusting for age, gender, and disease status. This was said to be the first report of an association of ASIP with specific human pigmentation characteristics.[123] Gudbjartsson *et al.,*[124] studied 2,121 individuals with cutaneous melanoma and 2,163 individuals with basal cell carcinoma, and over 40,000 controls. A two-SNP haplotype near the ASIP gene was the variant most strongly associated with both cutaneous melanoma and basal cell carcinoma.[23,124] ASIP is a promising melanoma susceptibility candidate as ASIP antagonizes the binding of MSH to MC1R and stimulates the production of pheomelanin.[124] It remains to be investigated whether the interaction of MC1R and ASIP can enhance prediction of human pigmentation and melanoma risk.[123]

#### TYR

Tyrosinase (TYR; CUTANEOUS MALIGNANT MELANOMA SUSCEPTIBILITY TO, 8; CMM8; gene map locus 11q14-q21) catalyzes the first two steps, and at least one subsequent step, in the conversion of tyrosine to melanin.[125]

Gudbjartsson *et al*.,[124] reported that a coding sequence variant in TYR(R402Q) showed the second most significant association with cutaneous melanoma and basal cell carcinoma.[23,124] A study reported that when tyrosinase and melan-A(ELANOMA ANTIGEN RECOGNIZED BY T CELLS 1; gene map locus Chr.9) were used together, 100% of the formalin-fixed, paraffin-embedded uveal melanoma samples tested positive for one of those markers. The same study concluded that the expression of melanocytic markers such as melan-A and tyrosinase is not influenced by radiotherapy or any clinico-pathological parameter.[126]

A tyrosinase peptide vaccine may stimulate the body's immune system to find and kill melanoma cells. Over 30 ongoing Phase I and Phase II RCTs are studying such vaccines.[127]

#### TYRP1

Gudbjartson *et al*., reported an SNP located near the TYRP1 (TYROSINASE-RELATED PROTEIN 1; TYRP; gene map locus 9p23) gene locus to be associated with an increased risk for melanoma.[124] Like TYR, TYRP1 DNA is also used in investigational vaccines to immunize melanoma patients against TYRP2.[128]

The variants in MC1R, ASIP, TYR and TYRP1 have been identified as independent low-penetrance susceptibility factors, but little is known about the combined risk of two or more factors.[2] Low-penetrance genes are associated with only a small increase in risk but contribute to the complex interrelation between pigmentation and sun-sensitivity phenotypes that interact with exposure to UV light to affect predisposition to melanoma.[129]

## CONCLUSIONS

**Figure 2:**
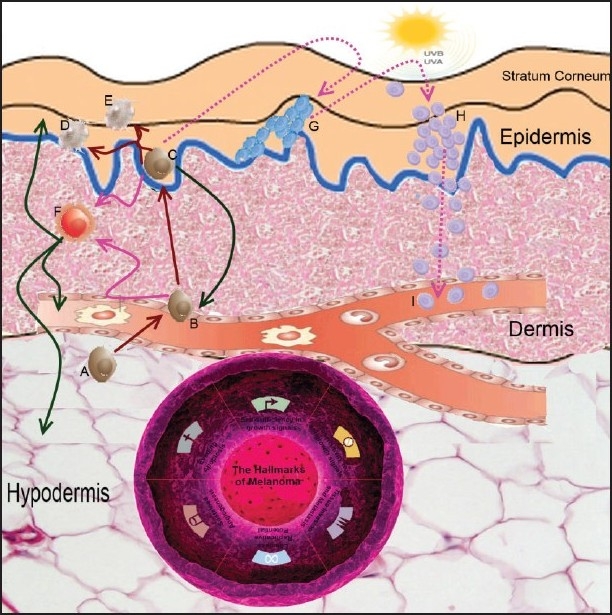
Melanoma: Stem cells, sun exposure and hallmarks of carcinogenesis. Melanoma stem cells undergo several genome changes, either mutations or epigenetic. Melanoma stem cells need to achieve growth self-sufficiency, the ability to suppress anti-growth signals, to avoid apoptosis, to replicate without limit, to invade, proliferate and sustain angiogenesis prior to malignancy development. In the epidermis, stem cells are still capable of acquiring new traits via changes to their genome. A: precursor melanocyte migrating toward the dermis. B and C: precursor melanocytes in the dermis and epidermis respectively. D and E: mature melanocytes in the epidermis and stratum corneum. F: Malignant melanocyte (melanoma stem cell). Malignant transition could be either due to spontaneous mutations (The Hallmarks of Melanoma) or –partially- due to UV-induced genome changes (either mutations or epigenetic). During this process which is anticipated to last several years, some precursor cells might acquire the ability to replicate, invoke own growth signals and avoid apoptosis, being capable of causing benign nevi (G). Further genomic instability, perhaps UV-induced, leads to the development of metastatic melanoma (H), which could cause bloodborne metastases (I)

We demonstrate that melanoma cannot be a “black box” for researchers anymore. Melanocyte precursor cells -stem cells more likely- undergo several genome changes, either UV induced or not. These changes could be either mutations or epigenetic. These changes provide the stem cells with the ability to self-invoke growth signals, the ability to suppress anti-growth signals, to avoid apoptosis, to replicate without limit, to invade, proliferate and sustain blood supply. Stem cells are able to collect progressively these changes in their genome. These -or some of these- new functional potentials, drive melanocyte precursors to the epidermis where they proliferate and might cause benign nevi. In the epidermis, they are still capable of acquiring new traits via changes to their genome. As time passes, such changes could add up to transform melanocyte precursors to malignant melanoma stem cells [Figure 2].

### Future pespectives for researchers and clinicians

Considerable effort is currently focused on patient selection on the basis of molecular profiling and on combination of drugs targeting melanoma-specific aberrations in signaling and apoptotic pathways to overcome the many resistance mechanisms in melanoma cells.[129] Pharmacogenomics deal with the influence of genetic variations on drug response in patients by correlating gene expression or SNPs with a drug's efficacy or toxicity. Pharmacogenomics aim to develop rational means to optimize drug therapy, with respect to the patients' genotype, thus to ensure maximum efficacy with minimal adverse effects.[130] Pharmacogenomics constitute a promising field with regard to melanoma patients. We reviewed a number of treatment approaches based on previous genomic profiling and reported relevant ongoing clinical trials.

Development of novel monoclonal antibodies is also another important field in future melanoma patient care. With growing knowledge of the etiopathogenesis of the disease, we investigate ways to block more molecular signals and impair more cellular metabolic paths.

Melanoma is one of the most immunogenic cancers, thus enhancement of host defense will be further studied in the future. New vaccines would be expected to enter clinical trials, while some of the vaccines already under trial could find their way into everyday oncology practice.

## AUTHOR'S PROFILE

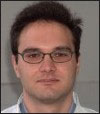



**Dr. Thrasivoulos-George Tzellos, MD** is a Fellow Researcher affiliated with the Department of Pharmacology, Aristotle University of Thessaloniki. His research interests include skin carcingenesis, skin ageing, drug metabolism and adverse effects, medical writing and systematic review methodology

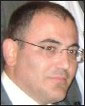



**Dr. Stefanos Triaridis, MD** is a lecturer in Otolaryncology - Head and Neck Surgery in Aristotle University of Thessaloniki. His research interests include head and neck surgical oncology, head and neck trauma, laser applications in surgery, medical research methodology and medical education.

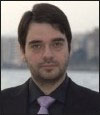



**Dr. A. Kyrgidis, MD** is a Fellow Researcher located in Thessaloniki Greece. His affiliations include, Aristotle University of Thessaloniki, Dpts of Oral Maxillofacial Surgery, OtoRhinolaryncology-Head & Neck Surgery and Pharmacology, Theagenio Cancer Hosptial. Dpt Maxillofacial Surgical Oncology. Athanassios Kyrgidis, MD, is versed in inflammatory and metabolic bone diseases, head and neck cancer, skin cancer and trauma. His areas of research expertise are in the epidemiology and pathophysiology of both osteonecrosis of the jaws and in carcinogenesis. His publications include the first evidence based association of dental extractions and dentures with osteonecrosis of the jaws; new proposals and interventions for preventing osteonecrosis of the jaws; the identification and description of risk factors for non melanoma skin cancer; esophageal carcinoma and melanoma; medical writing methodology.
